# Supplementary material for: Female community health volunteers’ experience in navigating social context while providing basic diabetes services in western Nepal: Social capital and beyond from systems thinking
Source: PLOS Glob Public Health. 2023 Nov 22;3(11):e0002632. doi: 10.1371/journal.pgph.0002632 (PMC10664953; doi:10.1371/journal.pgph.0002632)
Supplement: S1 Text — (DOC) [file pgph.0002632.s001.doc]

**S1 Text: Focus group discussion with FCHV (English version)**

**Date: ........................... Venue: ...................................... Time: ..........................................**

**Name of Moderator: ............................................ Name of Note taker: ............................**

**Number of participants: .....................................**

| **General information of FCHVs** | **1** | **2** | **3** | **4** | **5** | **6** | **7** |
| --- | --- | --- | --- | --- | --- | --- | --- |
| Age |  |  |  |  |  |  |  |
| Ward number |  |  |  |  |  |  |  |
| Educational Status |  |  |  |  |  |  |  |

**Setting a context:**

1. What kind of work do you generally do in the diabetes intervention?
2. How do you feel about this diabetes intervention so far?
3. How confident do you feel about your performance including technical skills?
4. What do you like the best about this program?

**Understanding the phenomenon (Opportunities and Challenges):**

1. Tell us about what motivates you to work in this program. Could you please give us some examples?
2. Have you had any experience while providing information related to diabetes was inconsistent with the religion/culture of service takers which are difficult for them to put into practice? *(****Probe but do not mention the terms such as*** *“drinking alcohol, smoking, eating non-vegetarian foods in more amount and bodyweight linked with wealthiness of family”)*
3. To perform your role, how supportive have you found the ….?

- Community people
- Community leaders
- Your family members
- Healthcare workers

1. How do you find the supervision and monitoring you have received till now?
2. What kinds of factors have affected you to carry out your diabetes related activities? (HINT: geographical/seasonal/transportation)
3. How is this program related to logistics issues with you?
4. Tell us about the challenges that you have faced while providing the services in the T2D intervention program. Examples?
5. How have you coped with the overall challenges you faced? *(use the examples from other questions above provided by them in the interview)*
6. How do you feel with your current?

- Workload?
- Working conditions?

1. Based on your experience, could you please tell us if there is anything that needs to be improved in this program? How can those things be improved?

**Closing Remarks:**

1. Does anyone have anything else that you’d like to say, perhaps something that we did not get to talk about?

*[If so, pursue as time allows]*

*[If not, thank and dismiss]*

*(I would like to thank you all for your active participation in today’s discussion.)*
