# Supplementary material for: Female community health volunteers’ experience in navigating social context while providing basic diabetes services in western Nepal: Social capital and beyond from systems thinking
Source: PLOS Glob Public Health. 2023 Nov 22;3(11):e0002632. doi: 10.1371/journal.pgph.0002632 (PMC10664953; doi:10.1371/journal.pgph.0002632)
Supplement: S2 Text — (DOC) [file pgph.0002632.s002.doc]

**S2 Text: In-depth Interview with FCHV (English version)**

**Date: ........................... Venue: ...................................... Time: ............................................**

**Name of Interviewer: ............................................ ID of participant: .........................**

| **General information of FCHVs** |  |
| --- | --- |
| Age |  |
| Ward no. |  |
| How long have you been working as an FCHV? |  |
| How long have you been working in the diabetes intervention? |  |
| Were you familiar with diabetes before this intervention? (yes/no) |  |
| Do you have experience working with other intervention programs on NCDs? |  |

**Setting a context:**

1. Tell me about how you became an FCHV?
2. Can you tell me about how you came to engage with the diabetes intervention? How do you feel about it?

**Understanding the phenomenon: + Clarifying the phenomenon:**

*(More positive experience)*

1. Tell me about the typical activities you perform in a diabetes intervention.
2. What incidents stood out particularly for you?
3. How did such an experience affect you? What changes did such experiences make to you (in your life in general)?
4. Can you describe any moment about ‘the situation favoring you’ or something that made it really easy to carry out your tasks? Can you explain how you felt and acted?
5. Can you tell me what aspects you would like to continue in this program? Why?
6. Tell me what makes you really happy/motivated/satisfied to continue working on this program.

*(More unpleasant experience)*

1. Tell me about what really did not work for you? What annoyed/unsatisfied you, what are the things that were not helpful?
2. How did such experiences affect you? What changes did such experiences make to you?
3. Can you describe any moment where you had a difficult time? Can you explain how you felt and acted? *(Based on the answer, ask what would make the situation different than she experienced)*
4. What would change such a situation? What would you like to add more? What would make it easier to perform your tasks?
5. What aspects would you omit? why?
6. Did any of your unpleasant experiences have affected your life in general? Would you say, there is a way out? How?

**Closing Remarks:**

1. Do you have anything else that you’d like to say, perhaps something that we did not get to talk about?

*[If so, pursue as time allows]*

*[If not, thank and dismiss] (I would like to thank you for your contribution to this interview.)*
