# Supplementary material for: Female community health volunteers’ experience in navigating social context while providing basic diabetes services in western Nepal: Social capital and beyond from systems thinking
Source: PLOS Glob Public Health. 2023 Nov 22;3(11):e0002632. doi: 10.1371/journal.pgph.0002632 (PMC10664953; doi:10.1371/journal.pgph.0002632)

# **S1 Table**

# **Study participants’ demographic profile**

*Table 1: Cross-tabulation between age and educational level of study participants (FCHVs)*


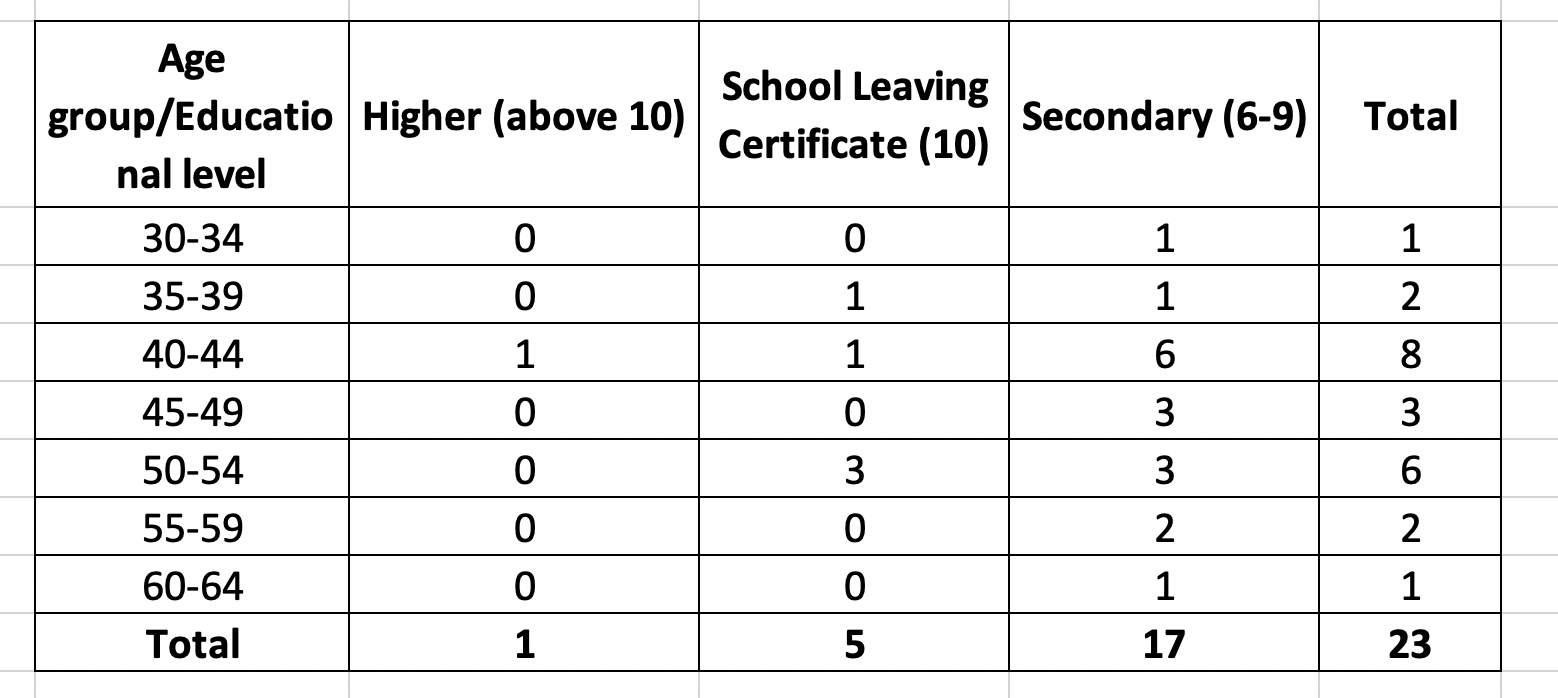

Supplement: S1 Table — (DOC) [file pgph.0002632.s003.doc]
