# Supplementary material for: Female community health volunteers’ experience in navigating social context while providing basic diabetes services in western Nepal: Social capital and beyond from systems thinking
Source: PLOS Glob Public Health. 2023 Nov 22;3(11):e0002632. doi: 10.1371/journal.pgph.0002632 (PMC10664953; doi:10.1371/journal.pgph.0002632)
Supplement: Attachment — Submitted filename: Response to Reviewers.docx [file pgph.0002632.s004.docx]

27-07-2023

To

The Editor-in-Chief

PLOS Global Public Health

Submission of the manuscript entitled, ’Female Community Health Volunteers' experience in navigating social context while providing basic diabetes services in western Nepal: Social capital and beyond from systems thinking’

Referring to your letter dated 29^th^ May 2023, we would like to thank you for the thorough review provided by reviewers and for allowing us to submit the revised manuscript. We believe that the revised manuscript is significantly improved compared with the one we had initially submitted.

We have addressed the comments as follows: 1. ‘Reviewers’ comments (black colour font), and 2. ‘Authors’ responses (blue colour font). Please refer to the ‘Revised Manuscript with Track Changes’ for the revised manuscript with the changes made in a marked-up copy and a clean version of manuscript labelled ‘Manuscript’. We hope that the reviewers will be satisfied with the amendments which we have made to the manuscript, and that the manuscript will be considered for publication.

Yours sincerely,

Rekha

On behalf of the co-authors

**Response to Reviewers**

1. Structure the abstract according to the Journal requirement.

Response: Thank you for your suggestion. We have now restructured the abstract of the study as per journal requirement and also addressed reviewer’s comments. The word count of the abstract is now 258. which is according to the journal requirement. Please see page 2 for the revised text in the revised manuscript.

2. Clarify the purpose of the study.

Response: Thank you for your suggestion. The purpose of the study have now been clarified. The revised text (page 5, second paragraph) is:

*This study aims to explore the experiences of female community health volunteers (FCHVs) in a community-based pilot project in western Nepal, specially regarding the social challenges they face while delivering basic type 2 diabetes (T2D) services.*

3. Structure the description of the survey methodology and all its elements.

Response: Thank you for your comment. The study methodology has been well described and all its elements are also clearly listed and clarified, as per the journal guideline. In the revised manuscript, we have incorporated various topics, incuding the study design, study area, study participants and their recruitment strategy, study tools and techniques, data generation, data analysis, and ethical considerations. Please see pages 5-9 in the revised manuscript.

4. Describe the tool, and explain if the tools were validated and how, include references.

Response: Thank you for your comment. The study tools and techniques are now clearly mentioned and explained in the revised manuscript. Reasoning on how the tools are validated have been described supported by appropriate references. Formulation of study tools and selection of such tools and techniques are also scientifically described with references. Please see page 7 second pargraph in the revised manuscript.

5. Elaborate how the qualitative data were analysed, provide the theoretical background, methods and tolls used.

Response: Thank you for your comment. Data analysis section was substantially revised as per the reviewers’ comments. We have now explained how the collected data was scientifically managed and analysed within the framework of relevant theory. We have provided the detailed information on how raw data were systematically managed using NVivo software and thematized. We have also discussed how we interpreted the data using Putnam’s and other various scholars’ Social Capital theory to interpret our study findings. Please see the text below and also refer to pages 8-9 in the revised manuscript.

*The data analysis in our study began with the first FGD, and continued in a sequential manner [38]. As the study progressed, we had the opportunity to improve and gather high quality data from subsequent FGD and interviews. All IDIs and FGDs were audio-recorded, transcribed, and translated into English. Subsequently, the research team read through the transcripts to become familiar with the data. Inductive content analysis was undertaken to describe and systematically organize the phenomena [39]. We interpreted both the verbal and non-verbal responses to give the meanings of the words during the latent content analysis [39][40]. Thereafter, we performed open coding using Nvivo software to identify patterns and arranged varied codes systematically [31]. Subsequently, sub-categories were freely generated and grouped by merging the identical ones under several categories to reduce the frequencies [39]. We eliminated the overlapped, repeated, unclear and irrelevant sub-categories to manage data [36]. Thereafter, we combined and thematized the categories with similar meanings from FGDs and IDIs.*

6. Explain the inductive coding procedure as a remedy for Putnam’s theory limitations.

Response: Thank you for your comment. We have now explained how inductive coding procedure made us realize the need for further explanations of our study findings through Putnam’s Social Capital Theory. Moreover, we also realized the Putnam’s theory limitations to explain our study findings especially in regard to power dynamics, hence we supplemented Putnam’s concept by other scholars, primarily, Coleman and Bourdieu to give a holistic meaning FCHVs’ phenomena on T2D intervention. Please see the text below and also refer to page 9 in the revised manuscript.

*As we explored sub-themes, our findings gradually formed a cohesive picture, revealing that the challenges faced by FCHVs in T2D intervention were primarily rooted in social phenomena, with power dynamics outweighing technical challenges. Consequently, we recognised the importance of delving deeper into sociological aspects to provide additional explanations. Following a comprehensive review of several sociological theories, we decided to employ Robert Putnam’s and other scholars' concepts of the social capital theory, which are widely recognised within the health sciences for understanding health related outcomes globally, to interpret, structure, and analyse the study's findings [41][42][43]. While Putnam's conceptualization of social network, social trust, and norms of reciprocity closely aligned with our findings, we acknowledged the limitations highlighted by other scholars such as Fine, Szreter and Woolcock, Song et al., and John [42][43][44][45]. To address these limitations, we supplemented Putnam's concept by incorporating the contributions of other scholars, primarily Coleman, Bourdieu and Szreter and Woolcock [46][47][44]. Social capital theory helped us to interpret the findings on the challenges closely associated with trust, social networks, norms and values [43] that FCHVs encountered while providing T2D services. We used the definitions of bonding, bridging, and linking social capital proposed by Szreter and Woolcock to explain the functioning of FCHVs' social networks, social trust, and norms of reciprocity in a more nuanced manner. Coleman's operationalization of resources facilitated our understanding of the actions of individuals and community members [46], while Bourdieu's conceptualization of power dynamics added a crucial perspective to the analysis of social capital [47]. Furthermore, the concept of systems thinking [48] helped us visualize and analyze the feedback loops among interconnected elements of social capital, including other dominant factors discovered during our analysis. We developed the systems diagrams using Vensim [49] and Kumu [50] softwares.*
